# Impact of agronomic practices on microbial diversity in brown-desert soil: insights from the Aksu region, Xinjiang

**DOI:** 10.3389/fmicb.2025.1522763

**Published:** 2025-04-08

**Authors:** Wenbo Guo, Yanbo Fu, Silayiding Simayi, Yunmeng Wen, Qingyong Bian, Jinquan Zhu, Zhigang Liu, Hanming Su, Yanhong Wei, Guohong Liu, Haifeng Li

**Affiliations:** ^1^Turpan Experimental Station, Xinjiang Uygur Autonomous Region Academy of Agricultural Sciences, Turpan, China; ^2^Agricultural Resources and Environment Research Institute, Xinjiang Uygur Autonomous Region Academy of Agricultural Sciences, Ürümqi, China

**Keywords:** brown-desert soil, agronomic practices, soil microorganisms, relative abundance, community structure

## Abstract

This study highlights how different agronomic practices reshape the microbial communities structure in brown-desert soils of Xinjiang’s Aksu region, with the goal of informing sustainable soil stewardship and agricultural strategies. Employing an L_9_ (3^4^) orthogonal array, we assessed the effects of different planting densities, irrigation levels, and fertilization strategies on the soil’s physicochemical properties, enzymatic activity, and microbial community composition. Our results highlight the dual role of fertilization: while it is the best strategy to increase agricultural productivity on low-fertility soils in the short term, excessive fertilization can have potentially detrimental effects. (1) it triggers salt accumulation and exacerbates salinization, and (2) it leads to an imbalance in C/N metabolism that inhibits microbial bioactivity. Through high-throughput sequencing, we identified significant shifts in the soil’s bacterial and fungal populations (e.g., Proteobacteria and Ascomycota) in response to agricultural interventions, with the type and extent of fertilization being pivotal to microbial diversity. Redundancy analysis revealed a significant interplay between soil microbial assemblages and underlying physicochemical attributes. This research underscores the necessity for judicious agronomic practices to maintain the delicate balance of microbial life within the soil, offering critical insights for the sustainable soil management of agricultural lands in Xinjiang.

## 1 Introduction

Soil microorganisms play a pivotal role in nutrient decomposition, uptake, and transformation (Fan et al., 2013), contributing to the material cycle and nutrient flow within soil ecosystems (Patel et al., 2015). The abundance of soil microbial species and community structure are crucial indicators for evaluating soil health and quality (Soong et al., 2019). However, these functions and indicators are sensitive to various soil environmental factors (Yang et al., 2023), such as soil nutrient content (Stark et al., 2012) and enzyme activity (Liu et al., 2021). There is a brown-desert soil type in the Aksu region of Xinjiang that has high ecological vulnerability (Ming et al., 2020) and requires scientific management and conservation measures to improve its stability and productivity. Thus it also highlights the need to study its microbial community.

Among the typical agronomic practices, planting density significantly influences the yield and economic viability of rapeseed. Optimal planting density harnesses the advantages of group cultivation and maximizes light energy utilization without affecting morphology (Ruo-Lin et al., 2023). It also leads to variations in the community performance of soil microorganisms (Zhang et al., 2014). Traditional irrigation methods often struggle with controlling water volumes, leading to uneven irrigation and significant water wastage (Wang Xue et al., 2022). Conversely, drip irrigation enables the real-time integration of water and fertilizers, ensuring their precise and quantitative delivery to the root zone. This method reduces nutrient leaching and improves the utilization of water and fertilizers (Ming-Da et al., 2019). The application of water and fertilizer can alter soil microbial communities (Hicks et al., 2020) by modifying nutrient conditions in the soil (Sradnick et al., 2013) and indirectly affect the physicochemical properties of the soil, including soil pH (Lammel et al., 2018). Fungi, being more sensitive to fertilization compared to bacteria, exhibit notable shifts in diversity when exposed to altered soil conditions (Youchao et al., 2022). Additionally, soil water content is another major factor that can influence soil physicochemical properties (Yu et al., 2013). In particular, efficient nitrogen fertilization management not only promotes high yields of rapeseed but also reduces production costs and improves the ecological environment (He et al., 2013). To date, many studies have investigated the effects of nitrogen fertilizer addition on soil microorganisms. However, research on brown-desert soils in the Aksu region of Xinjiang is still limited. The low fertility and highly saline brown-desert soils characteristic of this region provide unique conditions for studying the effects of agronomic practices on soil microbial communities. Our study examines how agricultural practices, including planting density, irrigation, and fertilizer application, affect soil microbes in this particular context. Clarifying these impacts is essential for developing sustainable agricultural strategies in arid regions. The results of this study may not only inform broader agricultural and ecological practices outside Xinjiang, especially in similar arid or semi-arid ecosystems. For example, the insights gained could guide the management of soil microbial communities in other areas facing similar challenges such as low soil fertility and high salinity.

In this study, we investigated the abundance and community structure of microbial species in brown-desert soil in the Aksu region of Xinjiang, focusing on their responses to various agronomic practices. We initially examined the effects of different planting densities, irrigation levels, and fertilizer application rates on soil physicochemical properties and enzymatic activity and soil microbial communities. Additionally, we explored the relationship between soil physicochemical properties and microbial communities across the treatments. The study addressed two primary questions: (1) What is the impact of agronomic practices on bacterial and fungal diversity? (2) What are the key soil factors shaping bacterial and fungal diversity in response to these agronomic practices? By examining the response of soil microorganisms to these practices, our goal was to enhance the understanding of their effects on soil quality in brown-desert soil farmland, ultimately supporting scientifically informed field management strategies.

## 2 Materials and methods

### 2.1 Experimental site and materials

**Figure 1:**
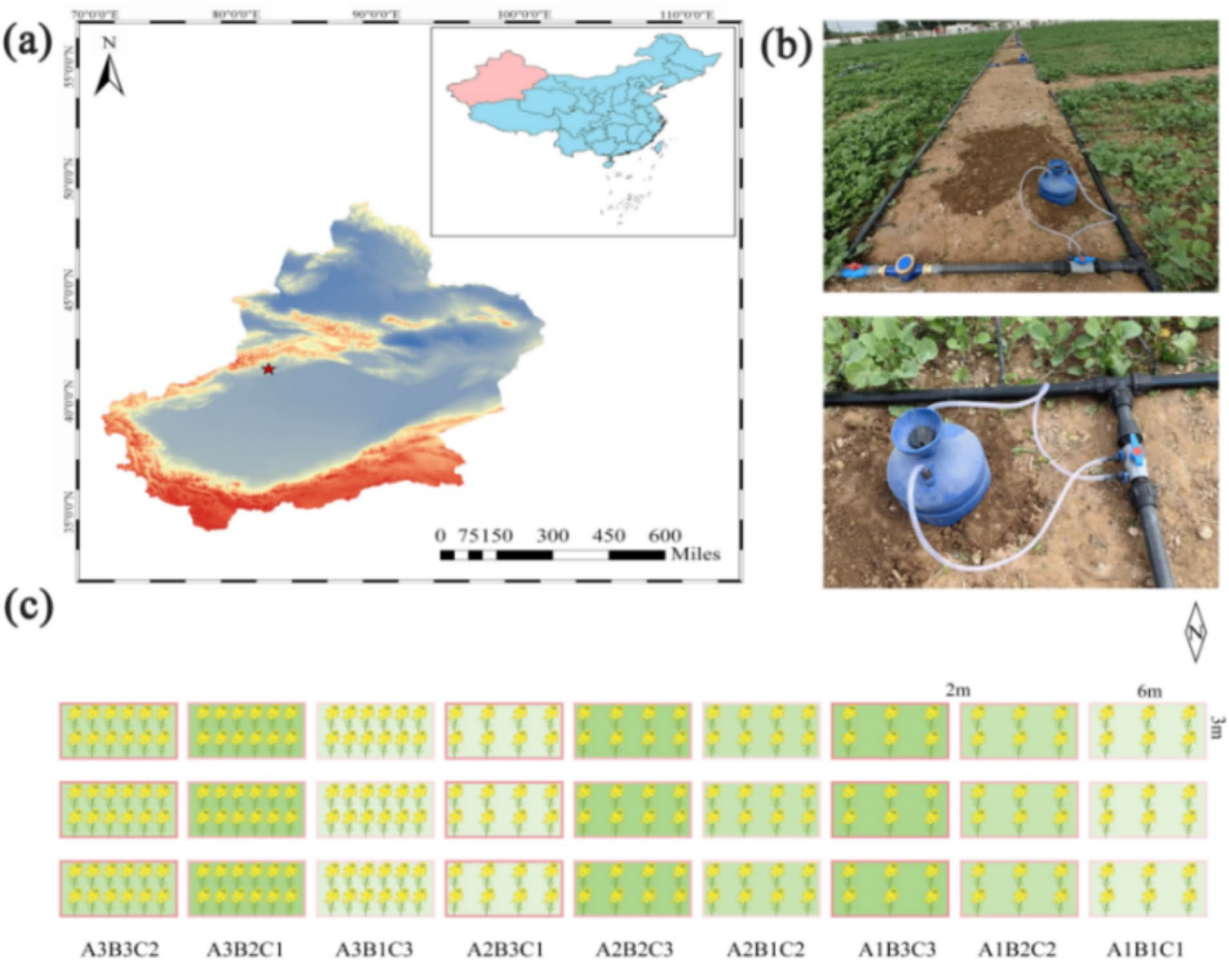
Experimental setup. **(a)** Experimental site located in Baicheng County, Aksu Region, Xinjiang Uygur Autonomous Region, China. **(b)** Water-fertilizer integration deployed at the experimental site. **(c)** The number of rapeseed in the box indicates planting density, the shade of green in the box fill indicates fertilizer application, and the shade of red in the border indicates the amount of water irrigation.

The experiment was conducted at the demonstration park of the Baicheng Agricultural Experiment Station, Xinjiang Academy of Agricultural Sciences, located in Baicheng County, Aksu Region, Xinjiang, China (Figure 1a). The test site coordinates were 41°79′N latitude and 81°87′E longitude, with an average elevation of 1,250 m. The area experiences a continental temperate arid climate, characterized by a frost-free period ranging from 133 to 163 days, an average annual precipitation of 171.13 mm, and an average annual temperature of 10.5°C (Supplementary Figure 1), and the prevailing wind is from the northwest, with an average wind speed of 2.5 m/s. The soil at the test site was classified as brown-desert soil, with an organic matter content of 27.84 g/kg, alkaline nitrogen at 55 mg/kg, available phosphorus (AP) at 38.2 mg/kg, and available potassium (AK) at 139 mg/kg. The soil had a pH of 8.28 and a conductivity of 329 μs/cm. The previous crop grown on the site was maize.

“Huayouza-62” is spring rapeseed (*Brassica napus* L.), a semi-wintering Polimar cytoplasmic male sterile line hybrid of the kale type. The complete life cycle of rapeseed is 140.5 days. It reaches a plant height of 157.1 cm and produces an average of 5.17 effective branches at any given time. Each plant hosts 231.2 effective angiosperms, with each angiosperm yielding 25.53 grains. The thousand-kernel weight is 4.11 g. The incidence rate of botrytis in Huayouza-62 is 17.75%, demonstrating robust resistance to inversion (Qian et al., 2021).

### 2.2 Experimental design

In order to involve more experimental factors and to obtain a greater amount of information while reducing the number of trials, we chose the L_9_ (3^4^) orthogonal experimental design (Roy, 2010). The test plot covered an area of 18 m^2^ (6 m × 3 m), with three replicates for each treatment. Sowing was conducted on April 12, 2023, using the manual strip sowing method. Density was assessed at the three- to five-leaf stage, with a row spacing of 20 cm. Three distinct rapeseed planting densities were tested: 300,000 plants/hm^2^, 600,000 plants/hm^2^, and 900,000 plants/hm^2^. Irrigation was applied four times during the reproductive period: 40 days after emergence, at the buds and shoots stage, during blooming, and at the junction stage. Drip irrigation was used, with a tube laid across four rows. Each plot was equipped with an independent water meter, and three water treatments were applied throughout the entire life cycle of the plants: 1,200 m^3^/hm^2^, 1,500 m^3^/hm^2^, and 1,800 m^3^/hm^2^. Fertilizer application was synchronized with drip irrigation, with each experimental plot connected to an independent fertilizer tank (Figure 1b). Three fertilizer treatments were implemented: 75 kg/hm^2^ urea +270 kg/hm^2^ slow-release fertilizer, 150 kg/hm^2^ urea +270 kg/hm^2^ slow-release fertilizer, and 225 kg/hm^2^ urea +270 kg/hm^2^ slow-release fertilizer (These experimental data were set up regarding local growing habits.). These treatments were labeled as A1B1C1, A1B2C2, A1B3C3, A2B1C2, A2B2C3, A2B3C1, A3B1C3, A3B2C1, and A3B3C2, according to the L_9_ (3^4^) orthogonal experimental design (Table 1;Figure 1c).

**Table 1:** Factor levels in the experimental design.

Treatment	A: Density (Plant/hm^2^)	B: Irrigation (m^3^/hm^2^)	C: Fertilization (kg/hm^2^)	D: Compound fertilizer (kg/hm^2^)
A1B1C1	300,000	1200	75	270
A1B2C2	300,000	1500	150	270
A1B3C3	300,000	1800	225	270
A2B1C2	600,000	1200	150	270
A2B2C3	600,000	1500	225	270
A2B3C1	600,000	1800	75	270
A3B1C3	900,000	1200	225	270
A3B2C1	900,000	1500	75	270
A3B3C2	900,000	1800	150	270

Urea (N: 46%), Compound fertilizer (N: P_2_O_5_: K_2_O = 12%: 13%: 25%).

### 2.3 Sample collection and determination methods

#### 2.3.1 Soil sample collection

In July 2023, soil samples were collected. To investigate the nutrient and microbial activity of the tillage layer and to make it better representative, we selected soil samples from 0 to 20 cm. Subsequently, the soil samples were passed through an 80-mm sieve to remove impurities and thoroughly homogenized. The homogenized soil samples were divided into three parts: One part was immediately treated with liquid nitrogen and stored at −80°C for 2 h for microbiological analysis, another part was stored at −5°C for soil enzyme activity analyses, and the remainder was air-dried under shaded conditions for soil physicochemical property analyses.

#### 2.3.2 Determination of yield and constitutive factors

Ten representative rapeseed plants were selected at the ripening stage to measure various parameters, including plant height, shape, branching pattern, number of primary and secondary effective branches, effective length of the main axis, number of effective angiosperms on the main axis, number of effective angiosperms on the entire plant, length and number of angiosperms, thousand kernel weight, and yield per plant. Subsequently, yield and constitutive factors were calculated (Lu, 2000).

#### 2.3.3 Analysis of soil physical and chemical properties

Soil pH, soil organic carbon (SOC), soil organic matter (SOM), total nitrogen (TN), total phosphorus (TP), total potassium (TK), available nitrogen (AN), AP, and AK were analyzed. Soil pH was determined using the potentiometric method (PH meter: BTYQ-BT920, accuracy: ±0.002). SOM was quantified via the volumetric method with potassium dichromate. SOC was calculated by dividing SOM by 1.724. TN was assessed using the acid digestion-indophenol blue colorimetric method (Ultraviolet–visible Spectrophotometer: HFD-FG-22, accuracy: 0.3 nm). TP was measured using the acid digestion-molybdenum antimony resistance colorimetric method. TK and AK were determined using acid digestion followed by flame atomic absorption spectroscopy (Kjeldahl Nitrogen Analyzer: D142570, accuracy: 1 μL; Flame atomic absorption spectrophotometer: RG-3604AA, accuracy: ±0.15 nm). AN was quantified using the potassium chloride leaching-indophenol blue colorimetric method. AP was analyzed using hydrochloric acid and ammonium fluoride leaching, combined with sodium bicarbonate leaching, followed by the molybdenum antimony resistive colorimetric method (Yuan et al., 2021).

#### 2.3.4 Soil enzymes

Urease, acid phosphatase, catalase, and sucrase have a close relationship with soil microorganisms. They are not only involved in the cycling of nitrogen, phosphorus, and carbon in the soil but also reflect the activity and diversity of soil microorganisms (Yanxiang et al., 2020). Therefore, they can be used as important indicators for assessing soil quality and the ecological environment. Urease (Item No: ml076938), acid phosphatase (Item No: ml076924), catalase (Item No: ml076930), and sucrase (Item No: ml076926) were assessed at Shanghai Enzyme Link Biological Company using enzyme-linked immunosorbent assay kits, following the manufacturer’s instructions. Purified antibodies against urease, acid phosphatase, catalase, and sucrase were employed to produce solid-phase antibodies via the double antibody sandwich method on a microtiter plate. Subsequently, urease, acid phosphatase, catalase, and sucrase were introduced into the microtiter wells containing the immobilized monoclonal antibodies, where they bound to horseradish peroxidase (HRP)-labeled antibodies specific to each enzyme, forming antibody–antigen–enzyme and HRP-labeled antibody–antigen–enzyme complexes. After thorough washing, color development was initiated by adding the substrate, 3,3′,5,5′-tetramethylbenzidine. The reaction was catalyzed by the HRP enzyme, resulting in a blue color that transitioned to yellow upon acid exposure. The intensity of the color was directly proportional to the concentrations of urease, acid phosphatase, catalase, and sucrase in the sample. Absorbance was measured at 450 nm using an enzyme marker.

The microbial carbon and nitrogen biomass were determined through potassium sulfate extraction and the fumigation-total organic carbon method. Microbial phosphorous biomass was determined using ammonium fluoride, hydrochloric acid/sodium bicarbonate extraction, fumigation, and the molybdenum-antimony colorimetric method (Yuan et al., 2021). The microbial C: N, C: P, and N: P ratios denote the relative proportions of carbon, nitrogen, and phosphorus within the microbial biomass.

#### 2.3.5 High-throughput 16S ribosomal RNA gene sequencing

Total genomic DNA was isolated from soil samples using the TGuide S96 Magnetic Soil/Stool DNA Kit (Tiangen Biotech [Beijing] Co., Ltd.) following the manufacturer’s instructions. The quality and quantity of the extracted DNA were examined via electrophoresis on a 1.8% agarose gel and quantified using the NanoDrop 2000 UV–Vis Spectrophotometer (Thermo Scientific, Wilmington, USA). The hypervariable V3-V4 region of the bacterial 16S rRNA gene was amplified using primer pairs 338F (5′-ACTCCTACGGAGGCAGCA-3′) and 806R (5′-GGACTACHVGGGTWTCTAAT-3′). The ITS region of the fungal 18S rDNA gene was amplified using primer pairs ITS1-1F-F: (5′-CTTGGTCATTTAGAGGAAGTAA-3′) and ITS1-1F-R (5′-GCTGCGTTCTTCATCGATGC-3′). Both forward and reverse 16S primers and ITS primers were tailed with sample-specific Illumina index sequences to facilitate deep sequencing. The polymerase chain reaction was performed in a total volume of 10 μL, comprising the DNA template (5–50 ng), forward primer (10 μM, 0.3 μL), reverse primer (10 μM, 0.3 μL), KOD FX Neo Buffer (5 μL), dNTPs (2 mM each, 2 μL), and KOD FX Neo (0.2 μL), with ddH_2_O added to make up the volume to 20 μL. The amplification protocol included an initial denaturation at 95°C for 5 min, followed by 20 cycles of denaturation at 95°C for 30 s, annealing at 50°C for 30 s, extension at 72°C for 40 s, and a final extension at 72°C for 7 min. Subsequently, the amplified products were purified using the Omega DNA Purification Kit (Omega Inc., Norcross, GA, USA) and quantified using the Qsep-400 (BiOptic, Inc., New Taipei City, Taiwan, China). Paired-end sequencing (2 × 250) of the amplicon library was performed on the Illumina Novaseq 6000 platform (Beijing Biomarker Technologies Co., Ltd., Beijing, China).

#### 2.3.6 Bioinformatic analysis

Clean reads then were conducted on feature classification to output an ASVs (amplicon sequence variants) by dada2 (Callahan et al., 2016), and the ASVs conuts less than 2 in all samples were filtered. Taxonomy annotation of the OTUs was performed based on the Naive Bayes classifier in QIIME2 (Bolyen et al., 2019) using the SILVA database (Quast et al., 2012) (release 138.1) with a confidence threshold of 70%. The Alpha diversity were calculated and displayed by the QIIME2 and R software, respectively. Beta diversity was determined to evaluate the degree of similarity of microbial communities from different samples using QIIME. Principal coordinate analysis (PCoA), heatmaps, UPGMA and nonmetric multidimensional scaling (NMDS) were used to analyze the beta diversity. Furthermore, we employed Linear Discriminant Analysis (LDA) effect size (LEfSe; Segata et al., 2011) to test the significant taxonomic difference among group. A logarithmic LDA score of 4.0 was set as the threshold for discriminative features. To explore the dissimilarities of the microbiome among different factors, a redundancy analysis (RDA) were performed in R using the package ‘vegan.’

### 2.4 Experimental data

Soil physicochemical property data were compiled and analyzed using WPS 2021. Subsequently, these data were subjected to the Shapiro–Wilk test, Levene test, and ANOVA, all performed by SPSS 27 statistical software. To gauge the intricacies of species diversity within each sample, alpha diversity analysis was conducted with the aid of QIIME2 software. Feature-level alpha diversity indices, such as observed OTUs, Chao1, Shannon, and Simpson indices, were calculated to estimate the microbial diversity within an individual sample. Redundancy analysis (RDA) was deployed to scrutinize the correlation between microbial communities and environmental factors, focusing on the relative abundances of microbial species across various phyla, and was executed using the R package ‘vegan.’ The construction of the corresponding network relied primarily on the ‘igraph’ package within R. Species interactions, inferred from relative abundances, were elucidated through Spearman’s rank correlation analysis, culminating in the development of a species interaction network visualized with R v4.3.2. Structural equation modeling (SEM) was based on Partial Least Squares Structural Equation Modeling (PLS-SEM), which was accomplished with the R V4.4.2 ‘plsem.’ package. Bacterial phenotype predictions were carried out on the BugBase platform, ecological function predictions were facilitated by the FAPROTAX database, and fungal phenotype predictions were conducted using the Fungi Functional Guild (FUNGuild). Lastly, the online platform BMKCloud was instrumental in the analysis of sequencing data.^1^

## 3 Results

### 3.1 Influence of agronomic practices on soil physicochemical properties

The results elucidated that the brown-desert soil in the Aksu region of Xinjiang exhibited low fertility and mild salinity. The main effect of fertilizer application was significant on electrical conductivity (EC), soil organic matter (SOM), soil organic carbon (SOC), total nitrogen (TN), total phosphorus (TP), available nitrogen (AN), available potassium (AK), urease and sucrase. In contrast, the main effect of irrigation was significant on pH, EC, TN, TP, AK, AP, urease, catalase, microbial biomass carbon (MBC), and microbial biomass nitrogen (MBN) were significantly affected. Comparatively, soil physicochemical properties, such as soil moisture content (SMC) and pH, were less influenced by fertilization and irrigation main effect. Fertilizer application increased SOM, SOC, TN, TP, and TK, and the effect of fast-acting fertilizers on soil nutrient content was more pronounced because the rapid release of fast-acting fertilizers may lead to leaching phenomena, resulting in nutrient imbalance. Additionally, nutrient contents were consistently higher at high planting density compared to low and medium planting densities [Soil salinity was measured based on EC value (us/cm)]. However, over-fertilization did not improve soil nutrient content, but rather exacerbated soil soluble salt accumulation, creating hypertonic environments and disrupting the balance of the soil microbial community. Regarding soil enzyme activity, both fertilization and irrigation significantly reduced the activity of urease. An increase in irrigation inhibited the activities of acid phosphatase, catalase, and sucrase, with irrigation exerting a greater influence than fertilizer application and planting density.

### 3.2 Effect of agronomic practices on bacterial and fungal community composition

Across all treatments, the bacterial composition was predominantly characterized by Proteobacteria (25.2%), Acidobacteria (20.2%), Gemmatimonadota (13.3%) and Actinobacteriota (8.5%), collectively constituting more than 60% of the composition. The results indicated a tendency for the relative abundance of Proteobacteria to increase with rising fertilizer application, although this trend was suppressed beyond a certain threshold. At high planting densities, Proteobacteria species exhibited a slightly higher abundance compared to low and medium planting densities. Conversely, the abundance of Acidobacteria species was significantly influenced by the amount of fertilizer applied, with a significant increase in species abundance as fertilization levels increased. Meanwhile, Actinobacteriota species demonstrated a declining trend in abundance with increasing irrigation, observed across both low and high planting densities.

**Figure 2:**
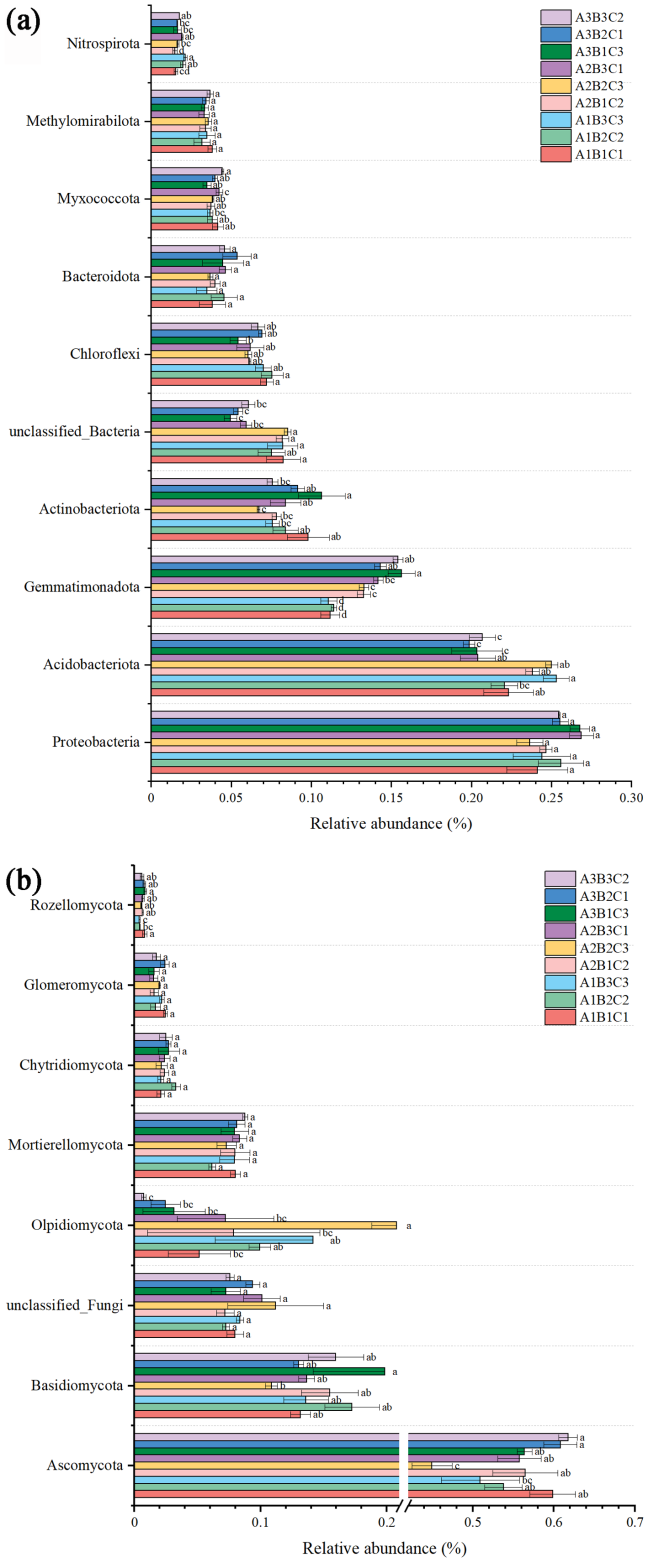
Histogram depicting the abundance of bacterial **(a)** and fungal **(b)** species.

The fungal composition was primarily dominated by Ascomycota (55.4%), followed by Basidiomycota (14.7%), unclassified fungi (8.4%), and Olpidiomycota (8.2%). The results indicated a significant influence of fertilizer application on the abundance of Ascomycota, which declined with increasing fertilizer application across various planting densities and irrigation levels. In contrast, the abundance of Basidiomycota initially increased and then declined with higher fertilizer application at low and medium planting densities, whereas a decrease was observed with increasing fertilizer application at high planting density. Conversely, the abundance of Olpidiomycota appeared to be more closely associated with irrigation, increasing with higher irrigation levels at low planting density and decreasing at high planting density. Overall, planting density, irrigation level, and fertilizer application did not affect the structure of bacterial and fungal communities in brown-desert soils. However, fertilizer application had an effect on bacterial and fungal abundance, with a more pronounced effect on fungi due to functional redundancy in bacteria (Figure 2).

### 3.3 Analysis of the alpha diversity index of bacteria and fungi under different agronomic practices

**Figure 3:**
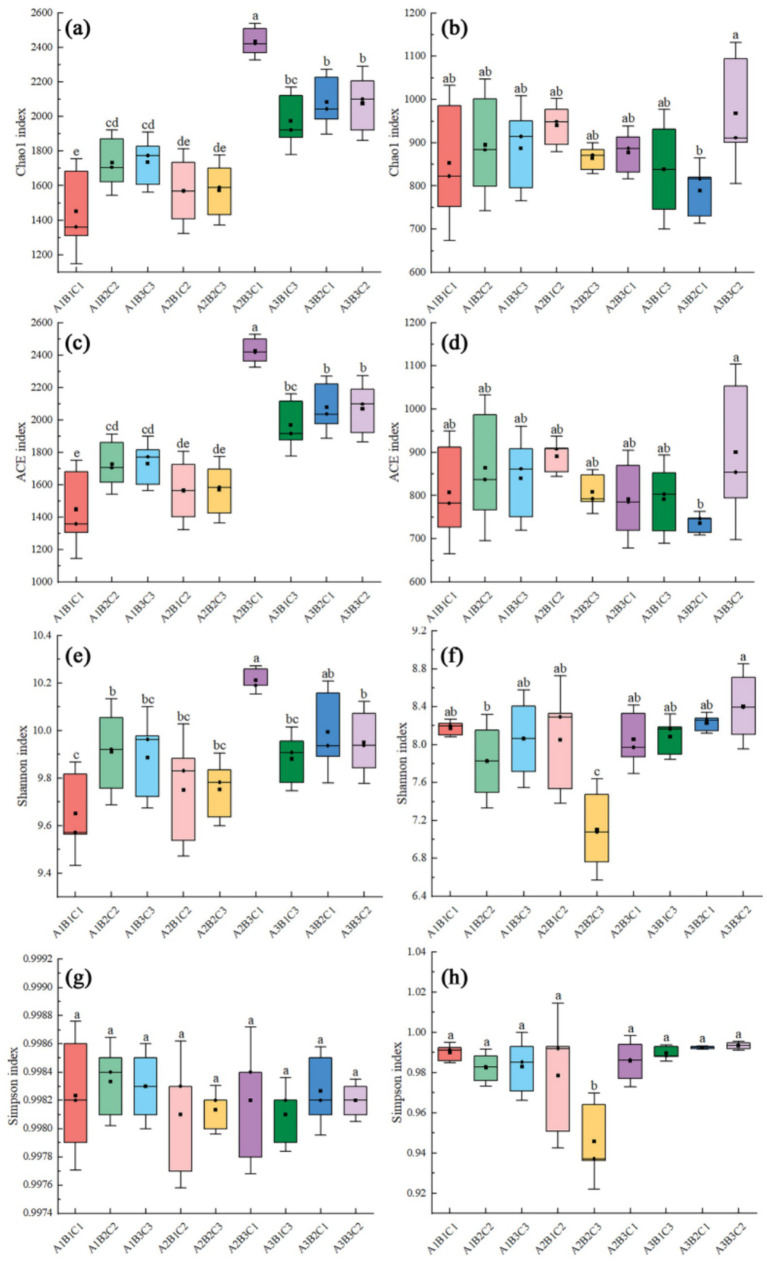
Box line plot illustrating inter-group differences in Alpha diversity indices of bacteria and fungi. **(a,b)** are Chao 1 indices for bacteria and fungi; **(c,d)** are ACE indices for bacteria and fungi; **(e,f)** are Shannon indices for bacteria and fungi; **(g,h)** are Simpson indices for bacteria and fungi.

Planting density, irrigation, and fertilizer application influenced bacterial and fungal alpha diversity. The results showed that fertilizer application significantly affected the bacterial OTU count, with over-fertilization leading to a decrease in Chao1 richness (Chao1), Abundance Coverage-based Estimator (ACE), and Shannon indices. Higher indices were observed at high planting density compared to low and medium planting densities, with the Chao1, ACE, and Shannon indices reaching their highest values under the A2B3C1 treatment. Furthermore, fertilizer application significantly influenced fungal OTUs. At low planting densities, over-fertilization reduced the Chao1 and ACE indices of the fungi but increased the Shannon index of the fungi, which could be attributed to the fact that over-fertilization led to soil acidification and salinization, which directly inhibited the growth of the fungi. Conversely, at medium and high planting densities, excessive fertilization reduced all fungal indices, with the Shannon index showing a more pronounced decrease. The Chao1, ACE, and Shannon indices for both bacteria and fungi were highest under the A3B3C2 treatment (Figure 3).

### 3.4 RDA of soil bacteria and fungi with soil physicochemical properties

**Figure 4:**
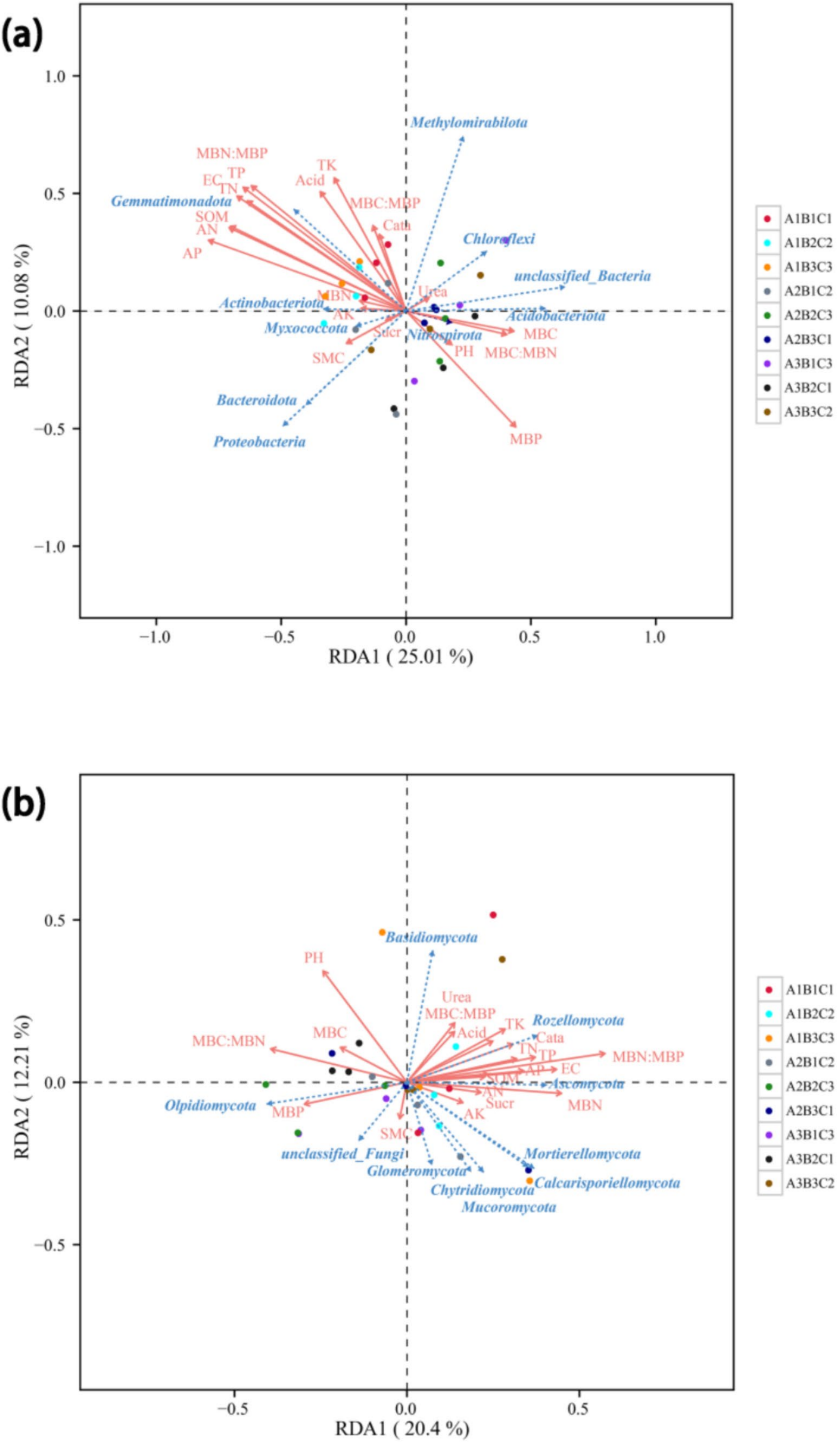
Redundancy analysis (RDA) of bacteria **(a)** and fungi **(b)**. RDA of the relative abundance of bacteria and fungi across various tillage treatments. SMC, soil moisture content; SOM, soil organic matter; SOC, soil organic carbon; EC, electrical conductivity; TN, total nitrogen; TP, total phosphorus; TK, total potassium; AN, available nitrogen; AP, available phosphorus; AK, available potassium; MBC, microbial carbon; MBN, microbial nitrogen; MBP, microbial phosphorus; Acid, acid phosphatase; Urea, urease; Cata, catalase; Suar, sucrase.

RDA revealed the relationship between soil microbial abundance and soil physicochemical properties. The first bacterial ordination (RDA1 axis) was primarily associated with SOM, AN, AP, TN, TP, MBP, and the MBN: MBP ratio. This axis explained 25.01% of the total variance. The phylum Gemmatimonadota was positively correlated with SOM, AN, AP, TN, and TP and negatively correlated with MBP because of its important role in the decomposition of organic matter. In contrast, Bacteroidota and Proteobacteria were mainly positively correlated with SMC because their metabolic activities were favored by sufficient soil water content. The first fungal ordination was linked to pH, the MBC: MBN ratio, and the MBN: MBP ratio, explaining 20.4% of the total variance. Rozellomycota is positively correlated with SOM, TN, TP, TK, catalase, and urease, which are mainly involved in soil organic matter decomposition and nutrient cycling. In addition, Ascomycota and Olpidiomycota were positively correlated with MBN and MBP, respectively, as they have important roles in nutrient cycling (Figure 4).

### 3.5 Correlation analysis of soil microbial species, soil physicochemical properties, and the alpha index

**Figure 5:**
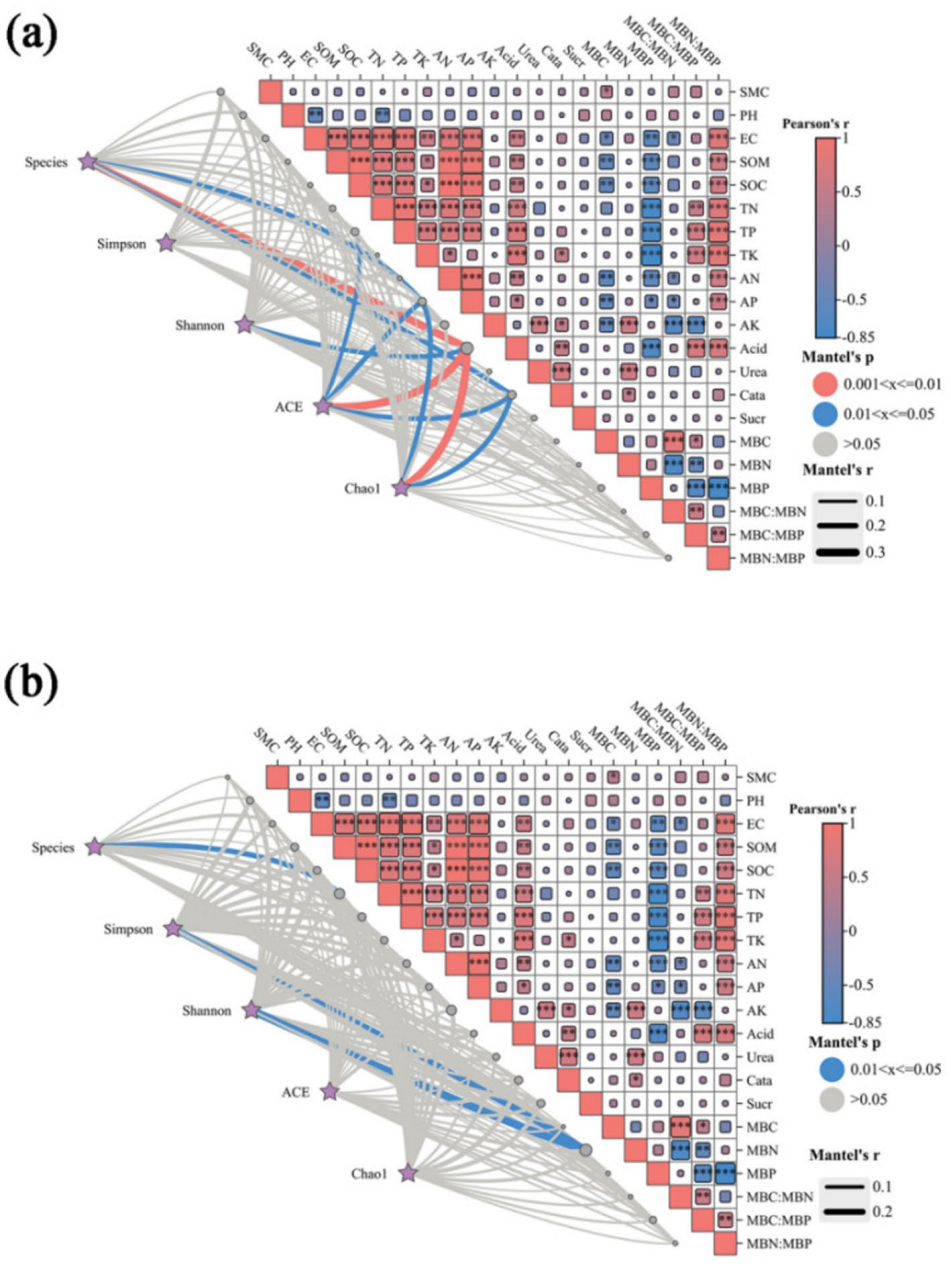
Species and environmental factors, alpha-correlation (Pearson) heatmaps, and network combination maps of bacteria **(a)** and fungi **(b)**. SMC, soil moisture content; SOM, soil organic matter; SOC, soil organic carbon; EC, electrical conductivity; TN, total nitrogen; TP, total phosphorus; TK, total potassium; AN, available nitrogen; AP, available phosphorus; AK, available potassium; MBC, microbial carbon; MBN, microbial nitrogen; MBP, microbial phosphorus; CN, MBC: MBN; CP, MBC: MBP; NP, MBN: MBP; Aci, acid phosphatase; Ure, urease; Cat, catalase; Sua, sucrase. “*” denotes significance at the 0.05 level, while “**” signifies significance at the “0.01” level. Mantel’s *P* is the *p*-value of Mantel analysis of environmental factors with species and the Alpha index; Mantel’s r is the *r*-value of Mantel analysis of environmental factors with species and the Alpha index; Pearson’s r is the *p*-value of correlation between environmental factors with species and the Alpha index. Heatmap in the upper right corner: correlation between environmental factors. Heatmap colors red and blue indicate positive and negative correlation, respectively, and the size of the heatmap block is consistent with the size of the correlation r. Bottom-left network diagram: the network relationship between species, Alpha index, and environmental factors. The color of the line is consistent with Mantel’s *P* in the legend, and the thickness of the line is consistent with Mantel’s r in the legend.

Soil microbial diversity was assessed based on OTU counts, Chao1 index, and Shannon index. The results showed significant positive correlations (*p* < 0.01) between SOM, SOC, tn, electrical conductivity (EC), AN, and AP; and significant negative correlations (*p* < 0.01) between MBP and SOM, SOC, TN, EC, AN, AP and acid phosphatase. In addition, there was a significant negative correlation between PH and both TN and EC (*p* < 0.05), and a highly significant negative correlation between MBC and MBP and SOM and SOC (*p* < 0.01): a highly significant positive correlation between the MBP ratio and SOM, SOC, EC, AN, AP, AK and acid phosphatase (*p* < 0.01). Acid phosphatase showed a highly significant positive correlation (*p* < 0.05) with SOM, SOC, EC, TN, TP, and TK, and catalase showed a highly significant positive correlation (*p* < 0.05) with TK, AK, acid phosphatase and urease. The abundance of bacterial genera showed a significant positive correlation with acid phosphatase (*p* < 0.05) and a negative correlation with AP and catalase (*p* < 0.05), while the diversity of bacterial genera showed a significant negative correlation with acid phosphatase (*p* < 0.05). The diversity of fungal genera was mainly negatively correlated with MBN (*p* < 0.05) (Figure 5).

### 3.6 Network analysis of soil bacterial and fungal communities

**Figure 6:**
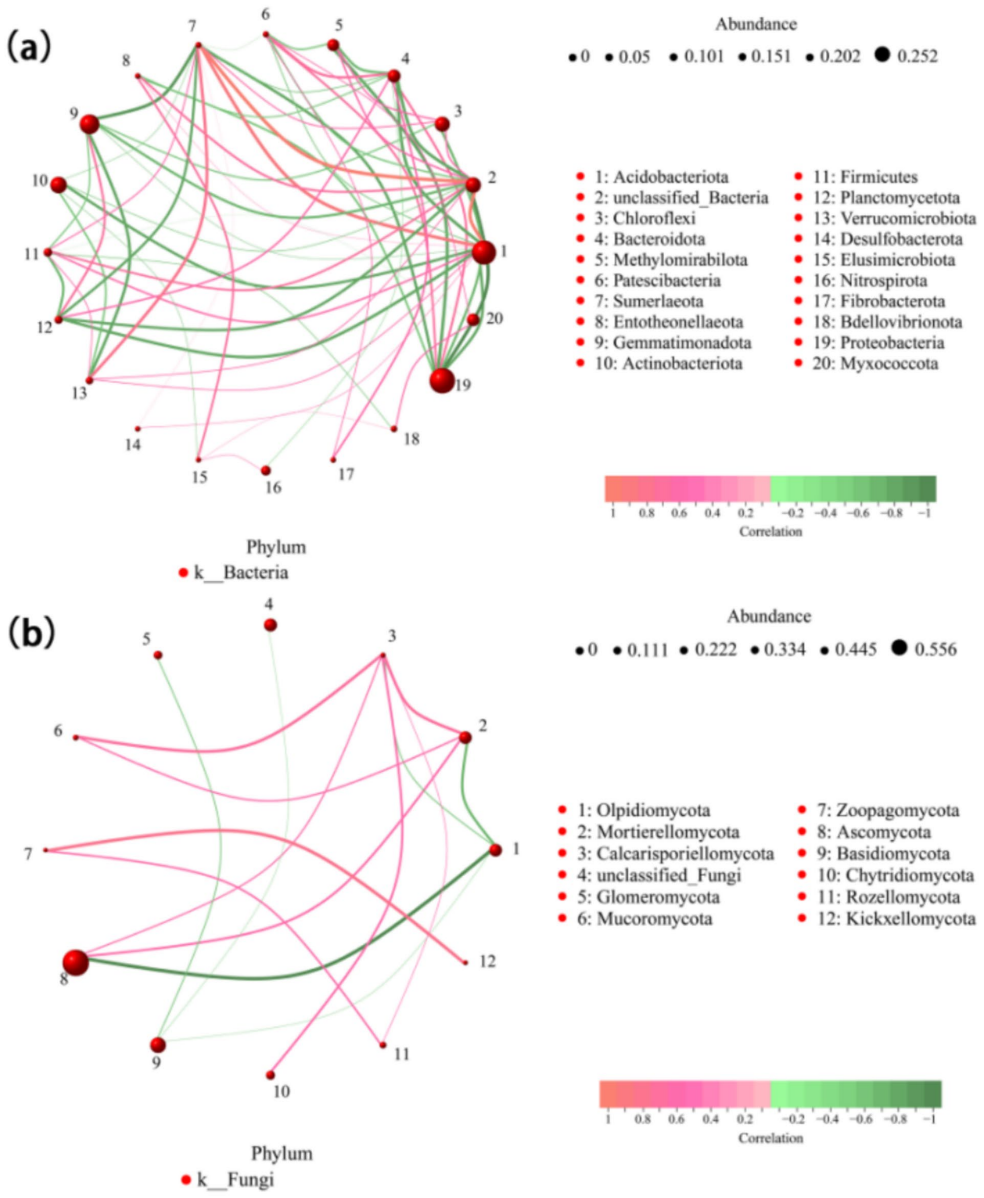
Relevance network diagram of bacteria **(a)** and fungi **(b)**. Network diagram illustrating bacterial community structures at the phylum level across different tillage conditions. Each circle represents a species, with the size of the circle proportional to the average abundance of the species. Lines represent correlations between species, with line thickness indicating the strength of the correlation. Red lines represent positive correlations, and green lines represent negative correlations.

A microbial community network was constructed to analyze interaction patterns in biological systems by correlating the horizontal abundance of soil microbial species at the phylum level. The horizontal species network graph of bacterial genera comprised 30 nodes and 100 edges, with a mean node degree of 0.667, a mean path length of 2.169, a network diameter of 11.28, a network graph density of 0.23, a clustering coefficient of 0.568, a mediator centrality of 0.104, and a modularity of 2.266. Conversely, the horizontal species network graph of fungal genera contained 41 nodes and 100 edges, with an average node degree of 4.878, an average path length of 2.979, a network diameter of 20.82, a network graph density of 0.12, a clustering coefficient of 0.378, a mediator centrality of 0.305, and a modularity of 0.31. Although the bacterial and fungal network graphs had a similar number of nodes and edges, the bacterial network exhibited higher density and clustering coefficient values, suggesting that bacterial connectivity is more complex, whereas fungal connectivity is relatively simpler (Figure 6).

### 3.7 BugBase phenotype prediction and FAPROTAX function prediction for bacteria

BugBase was used to normalize the OTUs based on predicted 16S rRNA gene copy numbers, and subsequently, microbial phenotypes were predicted using the provided pre-calculated files. Aerobic species accounted for 22% of the total bacterial community and were mainly associated with the decomposition of soil organic matter, influenced by planting densities and irrigation levels that limit gas exchange with the external environment. Anaerobic species accounted for 12.5% of the total bacterial community, with Gram-negative bacteria being the dominant phenotype, comprising 53.2%. These bacteria were susceptible to fluctuations, as increased fertilizer application correlated with a decrease in their population. Gram-positive bacteria represented 6.8% of the total bacterial community, with potentially pathogenic and stress-tolerant species comprising 2.6%. The biomass ratio between Gram-negative and Gram-positive bacteria exhibited a strong correlation with SOC levels, with Gram-negative bacteria prevailing.

**Figure 7:**
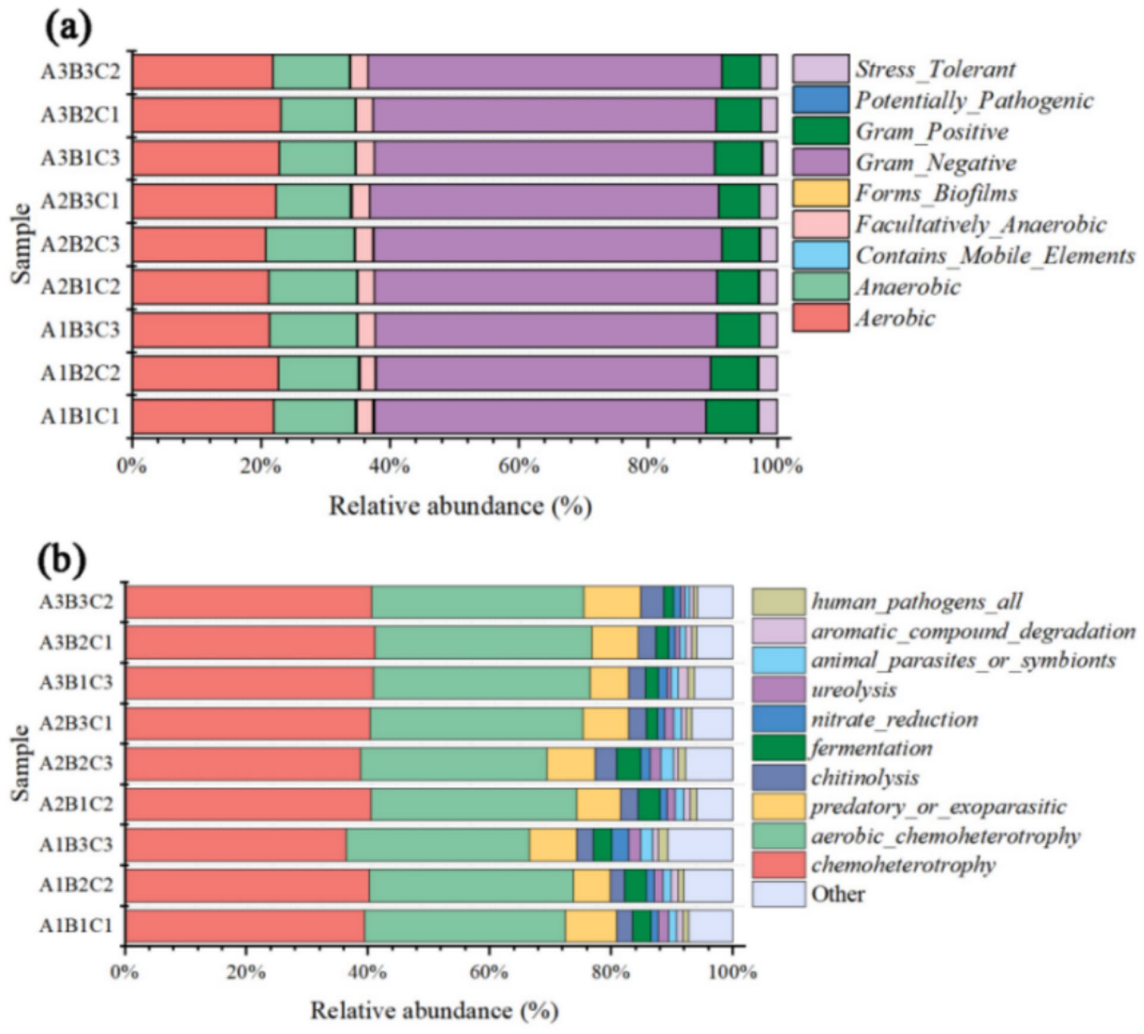
BugBase species histogram for bacteria **(a)** and bacterial FAPROTAX ecological function prediction **(b)**.

The FAPROTAX ecological function prediction revealed that chemoheterotrophs were the dominant functional bacterial group, comprising 40% of the total bacterial community, with aerobic chemoheterotrophs accounting for 33.6%. They are primarily involved in the cycling of nutrients such as nitrogen and sulfur in soil. Predatory or exoparasitic species constituted 7.6% of the community, while chitinolysis was observed in 3%. These results indicate that the dominant bacterial functional groups were chemoheterotrophs and aerobic chemoheterotrophs (Figure 7).

### 3.8 Fungal species prediction using FUNguild

**Figure 8:**
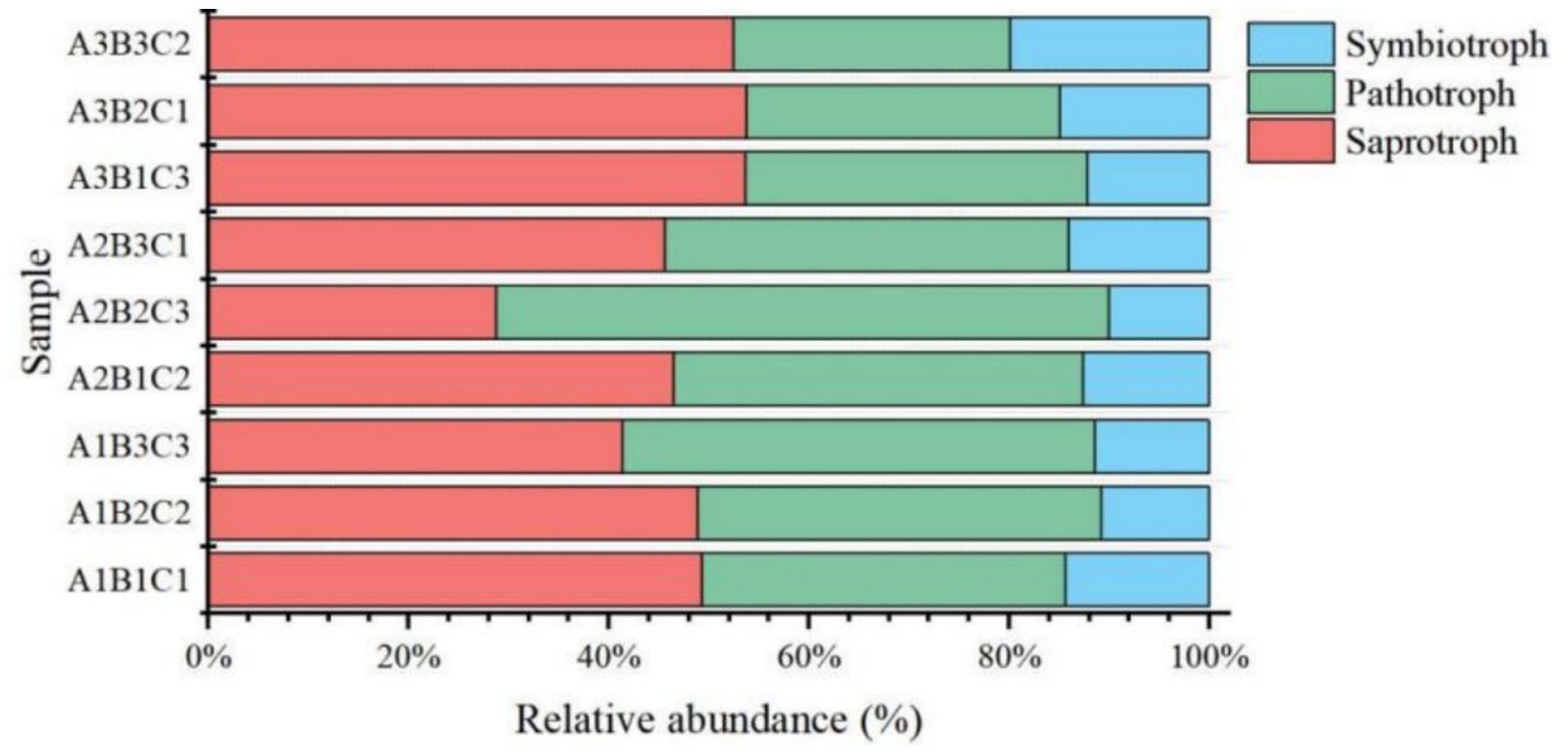
Histogram of fungi phenotypes predicted using FUNguild.

Based on the fungal phenotypic predictions by FUNGuild, fungi were classified according to their mode of nutrition into three categories: pathotrophs, which obtain nutrition by damaging host cells (including phagotrophs); symbiotrophs, which obtain nutrition by exchanging resources with host cells; and saprotrophs, which derive nutrition by degrading dead host cells. Pathotrophs accounted for approximately 46.7% of the fungi, symbiotrophs comprised 39.9%, and saprotrophs made up only 13.3%. Pathotrophs and symbiotrophs symbiosis with plants and engage in close nutrient exchange to promote plant growth and development, as well as to enhance crop resistance and adapt to various environmental stresses. Increased fertilizer application decreased the proportion of pathotrophs and symbiotrophs but increased the proportion of symbiotrophs. Among pathotrophs, Olpidiomycetes were the dominant fungal taxa (49.9%), followed by Glomeromycetes (33.6%) and Paraglomeromycetes (20.7%) within the saprotroph category. Conversely, Sordariomycetes (34.9%) constituted the primary fungal taxon among symbiotrophs (Figure 8).

### 3.9 Pathways of soil bacterial and fungal community diversity and composition

**Figure 9:**
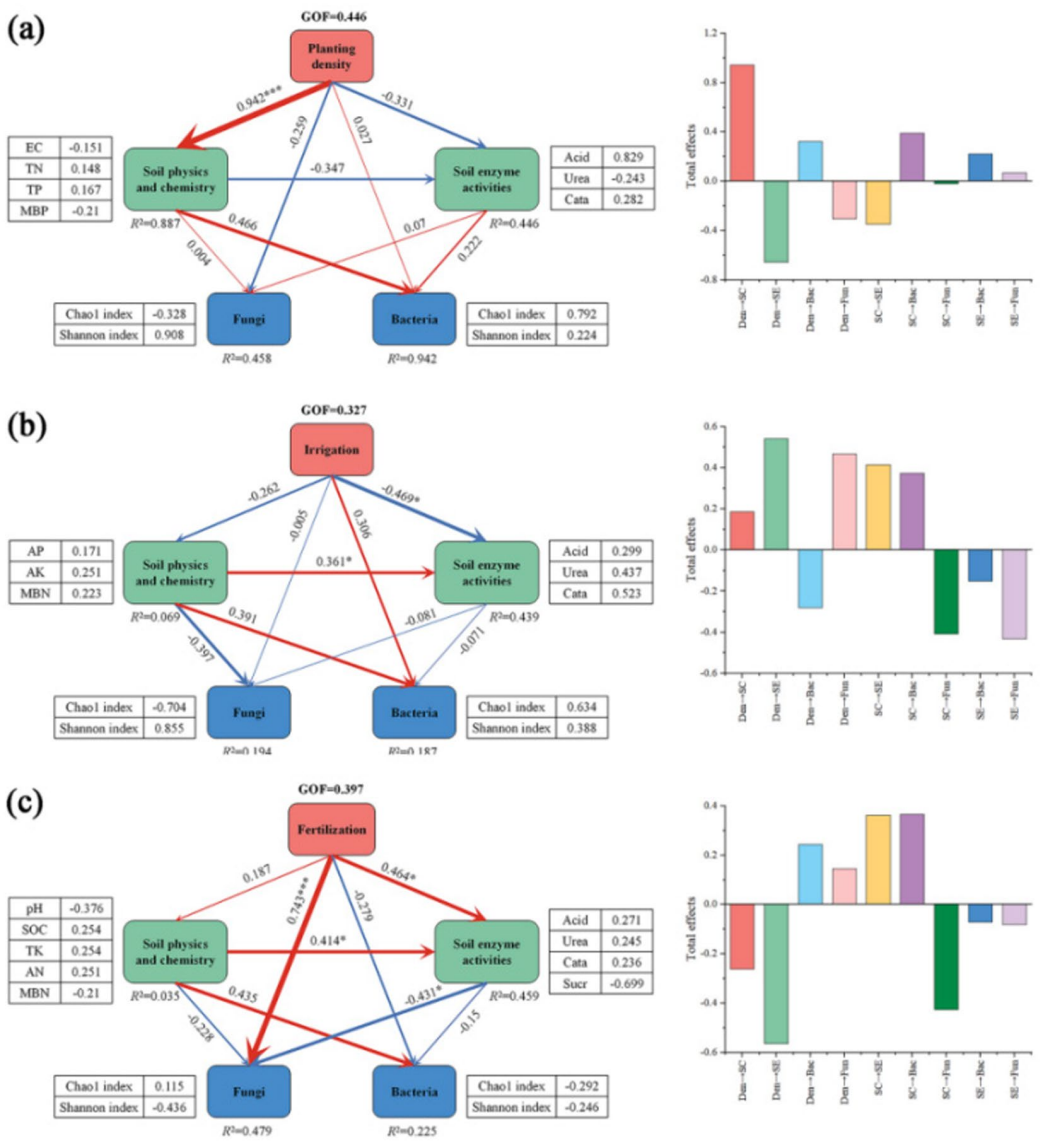
Effect of tillage treatments on the relative abundance and diversity of soil bacteria and fungi. **(a)** The effect of planting density and soil properties on the diversity and composition of soil bacterial and fungal communities. **(b)** The effect of irrigation volume and soil properties on the diversity and composition of soil bacterial and fungal communities. **(c)** The effect of fertilizer application and soil properties on the diversity and composition of soil bacterial and fungal communities. Partial least squares structural equation modeling (PLS-SEM) was used. The goodness of fit (GOF) index was employed to assess the overall degree of model fit, where a larger GOF value indicates a higher degree of model fit [GOF = 0.10, low degree of model fit; GOF = 0.25, medium degree of model fit; GOF = 0.36, high degree of model fit (Henseler and Sarstedt, 2013)]. SOM, soil organic matter; SOC, soil organic carbon; EC, electrical conductivity; TN, total nitrogen; TP, total phosphorus; TK, total potassium; AN, available nitrogen; AP, available phosphorus; AK, available potassium; MBC, microbial carbon; MBN, microbial nitrogen; MBP, microbial phosphorus; Acid, acid phosphatase; Urea, urease; Cata, catalase; Suar, sucrase; SC, soil physicochemical properties; SE, soil enzyme activities. Path coefficients denote the standardized predictive coefficients. Red indicates a positive pathway, and blue indicates a negative pathway. “*” denotes significance at the 0.05 level, while “**” signifies significance at the 0.01 level.

Structural equation modeling indicated that agronomic practices indirectly influence the diversity of soil bacteria and fungi by impacting soil physicochemical properties. Planting density indirectly affected the Chao1 index and Shannon index of bacteria through the positive effects of TN and TP and the negative effects of MBP and EC, while irrigation volume mainly negatively affected urease, acid phosphatase, and catalase, and fertilizer application not only directly positively affected the Chao1 index and negatively affected the Shannon index of fungi, but also indirectly affected the Chao1 index and negatively affected the Shannon index of fungi through the positive effects of urease, acid phosphatase, and catalase, and negatively affected sucrase. It also indirectly positively affects the Shannon index and negatively affects the Chao1 index of fungi by positively affecting urease, acid phosphatase, and catalase, and negatively affecting sucrase (Figure 9).

## 4 Discussion and conclusion

### 4.1 Effect of agronomic practices on soil physicochemical properties and soil enzyme activities

The present study provides specific insights into the intricate relationships between agronomic practices and the soil microbiome in the brown-desert soil of Xinjiang’s Aksu region. The results showed that the brown-desert soil in the Aksu region of Xinjiang exhibits low fertility and mild salinity. The main effect of fertilizer application was significant on electrical conductivity (EC), soil organic matter (SOM), soil organic carbon (SOC), total nitrogen (TN), total phosphorus (TP), available nitrogen (AN), available potassium (AK), urease and sucrase (Table 2). This finding aligns with previous studies (Manrubia et al., 2019; Jiao et al., 2016). However, fertilizer application had a significant dual effect (Table 2). Consistent with the results of previous studies (Wang et al., 2019; Ding et al., 2020), the excessive application of quick-acting fertilizers did not further increase the soil nutrient content, but increased soil salinization. Moreover, soil nutrient concentrations were higher at high planting densities than at low and medium planting densities (Table 2), likely due to dense plant lodging and inversion impacting light energy utilization efficiency in rapeseed, which subsequently reduced nutrient uptake.

**Table 2:** Effect of agronomic practices on soil physicochemical properties and analysis of variance.

Index	A1B1C1	A1B2C2	A1B3C3	A2B1C2	A2B2C3	A2B3C1	A3B1C3	A3B2C1	A3B3C2	Den	Irr	Fer
SMC (kg/m^2^)	19.5 (0.21) a	19.35 (0.10) a	19.43 (0.12) a	19.3 (0.04) a	19.39 (0.10) a	19.29 (0.02) a	19.29 (0.02) a	19.4 (0.01) a	19.48 (0.02) a	ns	ns	ns
pH	8.73 (0.03) a	8.7 (0.01) a	8.38 (0.09) b	8.38 (0.02) b	8.47 (0.02) b	8.43 (0.022) b	8.43 (0.12) b	8.57 (0.11) ab	8.37 (0.01) b	*	**	*
EC (μs/cm)	212 (2.31) h	193 (0.29) i	329 (0.87) f	350 (2.89) e	279 (1.44) g	381 (0.58) d	568 (2.02) a	427 (2.31) c	528 (2.02) b	**	**	**
SOM (g/kg)	19.5 (0.14) i	20.63 (0.06) h	21.56 (0.12) f	20.86 (0.03) g	24.82 (0.06) d	24.24 (0.03) e	31.62 (0.05) a	27.44 (0.06) b	25.2 (0.06) c	**	ns	**
SOC (g/kg)	11.31 (0.08) i	11.96 (0.03) h	12.5 (0.07) f	12.1 (0.02) g	14.4 (0.03) d	14.07 (0.02) e	18.34 (0.03) a	15.92 (0.03) b	14.62 (0.03) c	**	ns	**
TN (g/kg)	0.34 (0.03) e	0.23 (0.01) f	0.63 (0.01) d	0.62 (0.01) d	0.98 (0.00) c	0.97 (0.01) c	1.27 (0.03) b	1.25 (0.06) b	1.61 (0.01) a	**	**	*
TP (g/kg)	0.82 (0.01) f	0.69 (0.01) g	0.96 (0.01) de	0.82 (0.01) f	0.99 (0.01) d	0.94 (0.01) e	1.32 (0.01) c	1.4 (0.01) b	1.49 (0.01) a	**	**	*
TK (g/kg)	15.68 (0.06) c	11.62 (0.02) h	14.68 (0.06) e	14.43 (0.09) f	15.82 (0.06) c	12.63 (0.06) g	16.43 (0.12) b	15.42 (0.06) d	17.38 (0.06) a	**	ns	ns
AN (mg/kg)	37.55 (0.06) i	39.61 (0.06) h	41.17 (0.09) f	40.23 (0.08) g	48.41 (0.02) d	47.26 (0.07) e	62.44 (0.09) a	53.39 (0.06) b	48.68 (0.09) c	**	ns	**
AP (mg/kg)	17.77 (0.07) i	25.58 (0.06) e	23.44 (0.07) h	24.58 (0.05) g	24.82 (0.08) f	34.39 (0.02) d	50.67 (0.03) a	43.53 (0.06) b	34.77 (0.07) c	**	ns	ns
AK (mg/kg)	156.06 (0.04) b	135.42 (0.06) h	144.09 (0.32) f	151.42 (0.06) c	139.45 (0.12) g	145.6 (0.03) e	169.55 (0.06) a	147.42 (0.06) d	117.6 (0.03) i	ns	**	**
Acid (IU/L)	28.58 (0.3) f	27.21 (0.14) g	31.06 (0.29) e	33 (0.24) d	33.43 (0.26) d	21.52 (0.17) h	36.01 (0.23) c	39.21 (0.19) b	40.12 (0.37) a	**	ns	*
Urea (IU/L)	1019.04 (10.72) a	757.57 (4.67) c	823.28 (2.68) b	770.17 (1.94) c	625.92 (2.76) e	599.61 (3.45) f	1015.8 (7.83) a	557.44 (1.81) g	659.92 (2.1) d	**	**	**
Cata (U/mL)	7.55 (0.07) b	6.41 (0.02) e	7.63 (0.06) b	7.29 (0.04) c	5.86 (0.05) f	5.15 (0.05) g	8.03 (0.05) a	7.28 (0.07) c	6.84 (0.03) d	**	**	ns
Sucr (U/L)	853.09 (7.79) a	514.76 (5.19) f	685.58 (0.84) c	545.3 (2.71) e	575.63 (3.77) d	738.97 (5.69) b	500.27 (2.93) g	846.15 (4.55) a	551.84 (4.99) e	*	ns	**
MBC (mg/g)	22.77 (0.30) c	25.57 (0.30) b	26.74 (0.30) a	14.91 (0.30) f	17.19 (0.30) e	13.04 (0.30) g	12.45 (0.30) h	19.37 (0.30) d	23.09 (0.30) c	**	**	*
MBN (mg/g)	0.57 (0.01) a	0.43 (0.02) d	0.51 (0.01) c	0.58 (0.02) b	0.33 (0.01) f	0.55 (0.01) b	0.59 (0.01) a	0.44 (0.01) e	0.45 (0.01) e	ns	**	ns
MBP (mg/g)	0.45 (0.01) e	0.68 (0.01) a	0.57 (0.01) b	0.48 (0.01) d	0.29 (0.01) g	0.51 (0.01) c	0.37 (0.01) f	0.3 (0.01) g	0.26 (0.01) h	**	ns	ns
MBC: MBN	40.22 (0.03) d	59.51 (3.1) a	52.06 (0.5) b	25.9 (0.79) e	52.52 (0.09) ab	23.78 (0.33) f	21.2 (0.27) g	43.79 (0.76) c	51.72 (0.99) b	*	**	ns
MBC: MBP	51.19 (0.83) d	37.59 (0.06) f	47.09 (0.58) e	30.92 (0.52) h	58.54 (0.58) c	25.58 (0.45) i	34.03 (1.21) g	65.59 (1.38) b	90.32 (1.82) a	*	ns	ns
MBN: MBP	1.27 (0.02) c	0.64 (0.02) g	0.9 (0.02) f	1.2 (0.02) d	1.11 (0.02) d	1.08 (0.02) e	1.6 (0.02) b	1.5 (0.02) b	1.75 (0.02) a	**	*	ns

Soil physicochemical properties, soil enzyme activities, and microbial biomass of C, N, and P following oilseed rape harvest in August 2023.

SMC, soil moisture content; SOM, soil organic matter: SOC, soil organic carbon; EC, electrical conductivity; TN, total nitrogen; TP, total phosphorus; TK, total potassium; AN, available nitrogen; AP, available phosphorus; AK, available potassium; MBC, microbial carbon; MBN, microbial nitrogen; MBP, microbial phosphorus; Acid, acid phosphatase; Urea, urease; Cata, catalase; Suar, sucrase; Den, planting density; Irr, irrigation levels; Fer, fertilizer application.

Standard errors are indicated in parentheses. In cases where *p* < 0.05 (Kruskal-Wallis test), treatments with values denoted by different letters (e.g., a, b, and c) signify significant differences. “ns” denotes non-significance (*p* > 0.05). “*” denotes significance at the 0.05 level, while “**” indicates significance at the 0.01 level.

We also noted that both fertilizer application and irrigation significantly reduced urease activity, while increased irrigation inhibited acid phosphatase, catalase, and sucrase activities (Table 2). Notably, irrigation had a more pronounced effect on the activity of these enzymes than fertilizer application and planting density (Supplementary Tables 3, 4) because the catalytic mechanism of these enzymes is hydrolysis reactions (Yanxiang et al., 2020). These results contradict previous findings that slow-release fertilizers can enhance soil enzyme activities (Attademo et al., 2021; Teng et al., 2018). Soil enzymes may exhibit heightened sensitivity in low-fertilized and saline brown soil, where fertilizer application rates may exceed those typically reported in the literature (Gong et al., 2019). Excessive fertilization leads to changes in soil properties and suppression of soil microbial activity (Ji et al., 2014). But we need to explore the specific activity thresholds further. Additionally, the observed outcomes may result from interactions among planting density, irrigation levels, and fertilizer application.

SEM results suggest that soil properties can explain the effects of agricultural practices on soil microbial communities. In our results, planting density affected bacterial communities about TN and TP; irrigation and fertilizer application affected fungal and bacterial communities by altering urease, acid phosphatase, and catalase, and fertilizer application directly affected fungal communities (Figure 9). However, some studies differed (Ling et al., 2017), possibly related to differences in multifactorial experimental setups (including fertilizer type and amount). Thus, the mechanisms driving fungal and bacterial responses to agricultural practices may differ.

### 4.2 Effects of agronomic practices on soil microbial diversity and community structure

In this study, the predominant bacterial phyla in brown-deser soil were Proteobacteria (25.2%), Acidobacteria (20.2%), and Gemmatimonadota (approximately 13.3%) (Figure 3a). These bacteria were primarily associated with various soil physicochemical properties such as SOC, AP, EC, TN, TP, and pH (Figures 4, 5). The relative abundance of Proteobacteria increased following fertilizer application. Proteobacteria include nitrogen-fixing bacterial subclasses associated with elevated soil nitrogen content (Li et al., 2019; Dan-dan et al., 2024). However, over-fertilization inhibited this phenomenon, possibly as a result of unfavorable survival or microbial competition (Li et al., 2021) in high-nitrogen environments. Proteobacteria species abundance exhibited a slight increase at high planting densities compared to low and medium planting densities, positively correlating with elevated nitrogen levels under these conditions. Additionally, dense planting might have affected light and gas exchange with the external environment (Riping et al., 2022). Conversely, Acidobacteria species abundance was significantly affected by fertilizer application, exhibiting a significant correlation with AN, AP, and AK content in the soil. This was consistent with the observed significant increase in species abundance post-fertilization (Feng et al., 2021). In addition, Nitrospirota exhibited significant positive correlations with SOM, SOC, and AP (*p* < 0.05), highly significant positive correlations with AN, TN, and EC (*p* < 0.01), and highly significant negative correlations with pH and the MBC: MBN ratio (*p* < 0.01). Conversely, Myxomycophyta showed significant positive correlations with AN and AP (*p* < 0.05) (Figures 4, 5). Thus, agronomic practices influence the relative abundance and community structure of soil bacteria by modulating soil environmental conditions and nutrient profiles. We also noted that Gram-negative bacteria comprised 53.2% of the total bacterial community and exhibited a significant negative correlation with fertilizer application (Figure 7a). The biomass ratio of Gram-negative to Gram-positive bacteria was closely correlated with SOC levels, serving as an indicator of soil nutrient potential (Fanin et al., 2019). Additionally, aerobic bacteria, which accounted for 22% the total bacterial community, showed high sensitivity to planting density and irrigation levels because of direct impacts on the living environment of aerobic microorganisms, which inhibit their activities, such as high planting densities and soil compaction resulting from waterlogging (Songsong et al., 2019). FAPROTAX ecological function prediction (Figure 7b) identified chemoheterotrophs as the predominant functional bacterial group, representing 40% of the community, with aerobic chemoheterotrophy accounting for 33.6%. Fertilizer application significantly reduced this proportion, consistent with the findings of previous studies (Langenheder et al., 2010). Furthermore, the findings underscored a rich functional diversity (Langille et al., 2013), which ensures the stability of bacteria communities in response to external perturbations (Lin et al., 2022). These findings also confirmed the existence of functional redundancy within the bacterial community, with many metabolic pathways linked to genetic information and metabolism (Wang et al., 2018). Firstly, because of the higher species richness of bacteria, with a variety of metabolic abilities and survival forms leading to higher ecological diversity; and secondly, because of Microhabitat specialization, which creates a wide range of ecological niches based on resources and functions, which combined lead to a more complex network of bacterial communities (Zhang et al., 2024; Shepherd and Oliverio, 2024). Thus, microorganisms can adapt to environmental changes in response to minor environmental stresses, thereby maintaining ecosystem functioning.

Fungi exhibited greater sensitivity to the soil environment compared to bacteria, as evidenced by more pronounced changes in the relative abundance of fungi under each treatment, consistent with previous findings (Lin et al., 2022). Ascomycota emerged as the dominant taxon (55.4%), followed by Basidiomycota (14.7%), and Olpidiomycota (8.2%) (Figure 2b), primarily correlating with TN, TP, and TK (Figures 4, 5), aligning with previous studies (Li and Wu, 2018). Among these, Rozellomycota exhibited a significant positive correlation with pH (*p* < 0.05) and significant negative correlations with SOM, SOC, and TN (*p* < 0.05). Mucoromycota showed significant positive correlations with AK and acid phosphatase (*p* < 0.05), while Olpidiomycota demonstrated a significant positive correlation with TP (*p* < 0.05) (Figures 4, 5). In this study, fertilizer application significantly influenced the relative abundance of Ascomycota, which decreased with increasing fertilizer application across different planting densities and irrigation levels. Conversely, the abundance of Basidiomycota exhibited an increasing trend followed by a decrease with increasing fertilizer application at low and medium planting densities, consistent with previous studies (Chen et al., 2014). However, at high planting densities, Basidiomycota abundance showed a decreasing trend with increased fertilizer application, likely affecting gas exchange with the external environment. Olpidiomycota, in contrast, appeared more closely associated with irrigation, with its abundance increasing at low planting density and declining at high planting density with increasing irrigation levels (Wu et al., 2021; Figure 2b). Basidiomycota and Olpidiomycota have an important impact on ecosystem stability and function by promoting nutrient cycling and plant growth through the decomposition of organic matter, the formation of symbiotic relationships, and the improvement of soil structure (Manici et al., 2024). Overall, the quantity of fertilizer applied directly influenced soil physicochemical properties and enzyme activities, thereby affecting the relative abundance of soil microorganisms. Conversely, irrigation levels and planting density were more closely linked to limited gas exchange between soil microorganisms and the external environment. We also noted that based on the classification of nutritional modes, identified Olpidiomycetes as the predominant fungal taxon among pathotrophs (49.9%) (Figure 8). As a symbiotic nutrient type, Olpidiomycetes exhibits higher abundance in inter-root soils (Xiong et al., 2021). Meanwhile, Sordariomycetes emerged as the dominant fungal taxon among symbiotrophs (34.9%). As a member of Ascomycota, Sordariomycetes demonstrates a preference for degrading organic components and exhibits a positive correlation with nitrogen content within a specific range (Chen et al., 2012). Among saprotrophs, Glomeromycetes (33.6%) and Paraglomeromycetes (20.7%) play significant roles in decomposing plant residues and facilitating nutrient cycling (Egidi et al., 2019). This all emphasizes the functional resilience of these communities, which is essential for maintaining soil fertility and ecosystem services.

Overall, agricultural practices have different effects on soil microorganisms in brown-desert soils. Planting density, irrigation, and fertilizer application can all affect bacterial and fungal community structure and diversity by altering soil properties. However, fertilizer application has a more prominent dual role: rational application of fertilizer meets the nutrient requirements of the crop, whereas over-fertilization reduces soil microbial diversity and exacerbates salinization of brown-desert soils. In Xinjiang Aksu, the highest soil microbial abundance and diversity were found in brown desert-soil at planting densities of 600,000 plants/hm^2^, full-cycle irrigation of 1800 m^3^/hm^2^, and fertilizer application of 150 kg/hm^2^. However, this study is limited by time and space and may not be applicable to other regions, but it is crucial to maintain the ecological balance of the brown desert soil farmland in the Aksu region of Xinjiang in the short term. Therefore, in future studies, we will consider a wider range of geographical features and more functional experiments and apply them to similar arid or semi-arid ecosystems.

## Data Availability

The original contributions presented in the study are publicly available. This data can be found here: https://doi.org/10.6084/m9.figshare.28632369.v1.

## Footnotes

^1^ https://www.biocloud.net
