# Supplementary material for: Impact of agronomic practices on microbial diversity in brown-desert soil: insights from the Aksu region, Xinjiang
Source: Front Microbiol. 2025 Apr 8;16:1522763. doi: 10.3389/fmicb.2025.1522763 (PMC12011806; doi:10.3389/fmicb.2025.1522763)
Supplement: Supplementary file 1 [file Data_Sheet_1.docx]

Supplementary Material

# Supplementary Figures and Captions


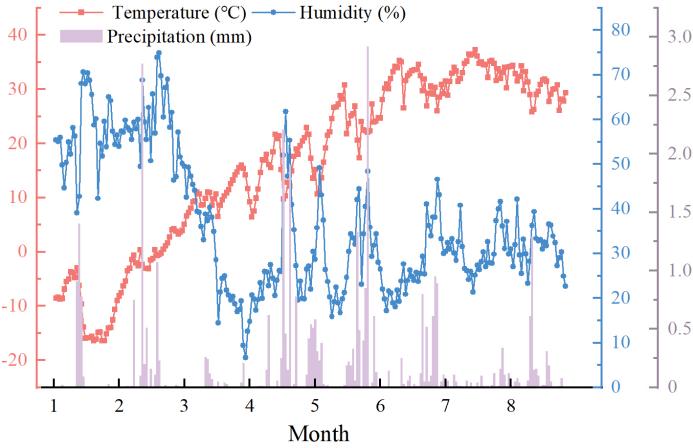


**Figure 1: Temperature and humidity at the experimental site.**

Meteorological data were sourced from WheatA Little Malt - Agrometeorological Big Data System (version 1.5.7b).

# Supplementary Tables

**Table 1. Critical fertility records of rapeseed under different agronomic practices.**

| Treatment | Sowing stage | Emergence stage | Pentaphyllous stage | Bloom stage | Bolting stage | Initial bloom stage | Blooming stage | Terminal stage | Ripening stage | Harvest stage | Growth period |
| --- | --- | --- | --- | --- | --- | --- | --- | --- | --- | --- | --- |
| A1B1C1 | 11 April 2023 | 4 May 2023 | 18 May 2023 | 31 May 2023 | 2 June 2023 | 12 June 2023 | 16 June 2023 | 3 June 2023 | 20 July 2023 | 5 August 2023 | 117 d |
| A1B2C2 | 11 April 2023 | 4 May 2023 | 18 May 2023 | 31 May 2023 | 2 June 2023 | 12 June 2023 | 16 June 2023 | 3 June 2023 | 20 July 2023 | 5 August 2023 | 117 d |
| A1B3C3 | 11 April 2023 | 4 May 2023 | 18 May 2023 | 31 May 2023 | 2 June 2023 | 12 June 2023 | 16 June 2023 | 3 June 2023 | 20 July 2023 | 10 August 2023 | 122 d |
| A2B1C2 | 11 April 2023 | 4 May 2023 | 18 May 2023 | 31 May 2023 | 2 June 2023 | 12 June 2023 | 16 June 2023 | 3 June 2023 | 20 July 2023 | 10 August 2023 | 122 d |
| A2B2C3 | 11 April 2023 | 4 May 2023 | 18 May 2023 | 31 May 2023 | 2 June 2023 | 12 June 2023 | 16 June 2023 | 3 June 2023 | 20 July 2023 | 13 August 2023 | 125 d |
| A2B3C1 | 11 April 2023 | 4 May 2023 | 18 May 2023 | 31 May 2023 | 2 June 2023 | 12 June 2023 | 16 June 2023 | 3 June 2023 | 20 July 2023 | 13 August 2023 | 125 d |
| A3B1C3 | 11 April 2023 | 4 May 2023 | 18 May 2023 | 31 May 2023 | 2 June 2023 | 12 June 2023 | 16 June 2023 | 3 June 2023 | 20 July 2023 | 15 August 2023 | 127 d |
| A3B2C1 | 11 April 2023 | 4 May 2023 | 18 May 2023 | 31 May 2023 | 2 June 2023 | 12 June 2023 | 16 June 2023 | 3 June 2023 | 20 July 2023 | 15 August 2023 | 127 d |
| A3B3C2 | 11 April 2023 | 4 May 2023 | 18 May 2023 | 31 May 2023 | 2 June 2023 | 12 June 2023 | 16 June 2023 | 3 June 2023 | 20 July 2023 | 18 August 2023 | 130 d |

**Table 2. Yield information and ANOVA of rapeseed under different agronomic practices.**

| Treatment | Bh  (cm) | Vl 1  （EA） | Vl 2  （EA） | Tot  （EA） | Ph  (cm) | St  (mm) | MIS  （EA） | Ml  (cm) | Den | SL  (mm) | Pn  （EA） | Sn  （EA） | TKW  (g) | PY  (g) | MP  (kg/hm^2^) |
| --- | --- | --- | --- | --- | --- | --- | --- | --- | --- | --- | --- | --- | --- | --- | --- |
| A1B1C1 | 70.7(9.53)ab | 4(1)ab | 0.7(0.67)b | 4.7(1.67)ab | 150.3(3.53)cd | 10.87(1.01)ab | 40(8.21)a | 51(7.55)a | 0.8(0.22)ab | 57.7(5.54)b | 26.4(0.44)a | 183.7(21.86)a | 4.288(0.12)ab | 20.7(2.12)ab | 2082 |
| A1B2C2 | 60.3(6.39)b | 6.3(0.88)a | 0.3(0.33)b | 6.7(1.2)ab | 153.7(4.81)bc | 10.13(0.22)ab | 39(8.89)a | 51.3(8.84)a | 0.87(0.04)ab | 65.07(5.99)ab | 27.7(1)a | 188.37(3.76)a | 4.4888(0.15)a | 23.4(0.96)a | 2631 |
| A1B3C3 | 79(8.39)ab | 4.7(1.2)ab | 0.3(0.33)b | 5(1)ab | 159(5.69)bc | 13.23(1.07)a | 57(2.08)a | 60.3(4.67)a | 0.86(0.02)ab | 54.6(0.95)b | 27(1.02)a | 223(29.21)a | 4.1438(0.08)ab | 24.7(2.33)a | 3108 |
| A2B1C2 | 90.3(7.75)a | 5.3(0.33)a | 1(1)ab | 6.3(0.88)ab | 169(4)ab | 10.84(0.29)ab | 58(1.67)a | 58.3(1.45)a | 1.06(0.07)a | 65.9(2.14)ab | 26.7(0.63)a | 194(11.72)a | 3.869(0.04)bc | 20(1.28)ab | 3326 |
| A2B2C3 | 70.7(5.36)ab | 2.7(0.67)b | 0.7(0.67)b | 3.3(1.33)b | 149(9.54)cd | 7.89(1.23)b | 43(2.4)a | 60.3(6.74)a | 0.64(0.14)b | 57.03(9.64)b | 25.2(1.76)ab | 207.4(11.84)a | 3.856(0.05)bc | 20.1(0.89)ab | 3744 |
| A2B3C1 | 70.7(3.93)ab | 4.7(0.88)ab | 1.3(0.33)ab | 6(0.58)ab | 141(7.09)d | 11.03(0.28)a | 54(5.03)a | 55.3(3.18)a | 0.85(0.08)ab | 64.2(4.4)ab | 25.4(0.47)ab | 175(11.53)a | 3.943(0.19)bc | 17.7(2.35)bc | 3069 |
| A3B1C3 | 81.7(3.18)ab | 4.3(0.33)ab | 3(0.58)a | 7.3(0.88)a | 160.7(9.84)bc | 10.8(0.55)ab | 57(7.64)a | 54.3(6.94)a | 0.98(0.02)a | 75.01(1.01)a | 24.5(0.37)ab | 173.4(6.33)a | 3.552(0.35)cd | 15.2(1.95)cd | 2745 |
| A3B2C1 | 96.7(12.84)a | 6(0.58)a | 0.7(0.67)b | 6.7(0.88)ab | 177.3(3.67)a | 12.96(1.23)a | 58(9.45)a | 58(3.06)a | 1.01(0.06)a | 61(3.42)ab | 24.7(0.45)ab | 178.7(15.68)a | 3.402(0.09)cd | 15(1.41)cd | 2630 |
| A3B3C2 | 81.7(11.05)ab | 5(0.58)ab | 2(1.15)ab | 7(1.53)ab | 171.7(5.55)ab | 12.25(1.38)a | 45(3)a | 66.3(13.93)a | 1.04(0.07)a | 60.58(6.06)ab | 22.9(1.39)b | 179(22.11)a | 3.263(0.05)d | 13.2(0.77)d | 1962 |
| Den | ns | ns | ns | ns | ** | ns | ns | ns | ns | ns | ** | ns | ** | ** | ** |
| Irr | ns | ns | ns | ns | ns | ns | ns | ns | ns | ns | ns | ns | ns | ns | ns |
| Fer | ns | ns | ns | ns | ns | ns | ns | ns | ns | ns | ns | ns | ns | ns | ns |

Bh: branch height; Vl 1: number of primary effective branches; Vl 2: number of secondary effective branches; Tot: total number of effective branches; Ph: plant height; St: stem thickness; MIS: number of effective siliques on the main inflorescence; MI: main stem length; Den: silique density; SL: silique length; Pn: Number of individual siliques; Sn: silique number; TKW: thousand kernel weight; PY: yield per plant; MP: yield. Superscripts not containing the same lowercase letters in the same column indicate significant differences (*P* < 0.05). "*" and "**" indicate significant differences at (*P* < 0.05) and (*P* < 0.01) levels, respectively, and ns indicates no significant difference.

**Table 3. Extreme range analysis of bacterial abundance and diversity across different agronomic practices.**

| Treatment | Factors | | | Chao1  index | Treatment | Factors | | | Shannon  index |
| --- | --- | --- | --- | --- | --- | --- | --- | --- | --- |
|  | A | B | C |  |  | A | B | C |  |
| A1B1C1 | 20,000 | 80 | 5 | 1788.419 | A1B1C1 | 20,000 | 80 | 5 | 9.880 |
| A1B2C2 | 20,000 | 100 | 10 | 1733.100 | A1B2C2 | 20,000 | 100 | 10 | 9.911 |
| A1B3C3 | 20,000 | 120 | 15 | 1736.514 | A1B3C3 | 20,000 | 120 | 15 | 9.887 |
| A2B1C2 | 40,000 | 80 | 10 | 1569.071 | A2B1C2 | 40,000 | 80 | 10 | 9.750 |
| A2B2C3 | 40,000 | 100 | 15 | 1573.917 | A2B2C3 | 40,000 | 100 | 15 | 9.752 |
| A2B3C1 | 40,000 | 120 | 5 | 2097.507 | A2B3C1 | 40,000 | 120 | 5 | 9.984 |
| A3B1C3 | 60,000 | 80 | 15 | 1974.431 | A3B1C3 | 60,000 | 80 | 15 | 9.881 |
| A3B2C1 | 60,000 | 100 | 5 | 2085.121 | A3B2C1 | 60,000 | 100 | 5 | 9.995 |
| A3B3C2 | 60,000 | 120 | 10 | 2076.354 | A3B3C2 | 60,000 | 120 | 10 | 9.950 |
| K1 | 5258.033 | 5331.921 | 5971.047 |  | K1 | 29.678 | 29.511 | 29.858 |  |
| K2 | 5240.495 | 5392.138 | 5378.524 |  | K2 | 29.486 | 29.658 | 29.612 |  |
| K3 | 6135.906 | 5910.375 | 5284.863 |  | K3 | 29.826 | 29.821 | 29.520 |  |
| k1 | 1752.678 | 1777.307 | 1990.349 |  | k1 | 9.893 | 9.837 | 9.953 |  |
| k2 | 1746.832 | 1797.379 | 1792.841 |  | k2 | 9.829 | 9.886 | 9.871 |  |
| k3 | 2045.302 | 1970.125 | 1761.621 |  | k3 | 9.942 | 9.940 | 9.840 |  |
| R | 298.470 | 192.818 | 228.728 |  | R | 0.113 | 0.103 | 0.113 |  |
| *P* | 0.087 | 0.128 | 0.110 |  | *P* | 0.996 | 0.996 | 0.996 |  |
| Sort | A>C>B | | |  | Sort | B>C>A | | |  |
| Optimum level | A3 | B3 | C1 |  | Optimum level | A3 | B3 | C1 |  |
| Optimal combination | A3B3C1 | | |  | Optimal combination | A3B3C1 | | |  |

A, B, and C denote experimental treatments; K1, K2, and K3 denote summation of identical treatments; k1, k2, and k3 denote mean values of identical treatments; R denotes range; and *P* denotes significance.The same is below.

**Table 4. Extreme range analysis of fungal abundance and diversity across different agronomic practices.**

| Treatment | Factors | | | Chao1  index | Treatment | Factors | | | Shannon  index |
| --- | --- | --- | --- | --- | --- | --- | --- | --- | --- |
|  | A | B | C |  |  | A | B | C |  |
| A1B1C1 | 20,000 | 80 | 5 | 853.491 | A1B1C1 | 20,000 | 80 | 5 | 8.175 |
| A1B2C2 | 20,000 | 100 | 10 | 894.929 | A1B2C2 | 20,000 | 100 | 10 | 7.826 |
| A1B3C3 | 20,000 | 120 | 15 | 886.826 | A1B3C3 | 20,000 | 120 | 15 | 7.729 |
| A2B1C2 | 40,000 | 80 | 10 | 940.791 | A2B1C2 | 40,000 | 80 | 10 | 8.052 |
| A2B2C3 | 40,000 | 100 | 15 | 864.239 | A2B2C3 | 40,000 | 100 | 15 | 7.104 |
| A2B3C1 | 40,000 | 120 | 5 | 877.524 | A2B3C1 | 40,000 | 120 | 5 | 8.058 |
| A3B1C3 | 60,000 | 80 | 15 | 838.477 | A3B1C3 | 60,000 | 80 | 15 | 8.083 |
| A3B2C1 | 60,000 | 100 | 5 | 788.821 | A3B2C1 | 60,000 | 100 | 5 | 8.229 |
| A3B3C2 | 60,000 | 120 | 10 | 968.650 | A3B3C2 | 60,000 | 120 | 10 | 8.405 |
| K1 | 2635.247 | 2632.760 | 2519.835 |  | K1 | 23.731 | 24.310 | 24.462 |  |
| K2 | 2682.554 | 2547.989 | 2804.371 |  | K2 | 23.214 | 23.159 | 24.283 |  |
| K3 | 2595.948 | 2733.000 | 2589.542 |  | K3 | 24.717 | 24.192 | 22.916 |  |
| k1 | 878.416 | 877.587 | 839.945 |  | k1 | 7.910 | 8.103 | 8.154 |  |
| k2 | 894.185 | 849.330 | 934.790 |  | k2 | 7.738 | 7.720 | 8.094 |  |
| k3 | 865.316 | 911.000 | 863.181 |  | k3 | 8.239 | 8.064 | 7.639 |  |
| R | 28.869 | 61.670 | 94.845 |  | R | 0.501 | 0.384 | 0.515 |  |
| *P* | 0.495 | 0.315 | 0.230 |  | *P* | 0.983 | 0.987 | 0.982 |  |
| Sort | C>B>A | | |  | Sort | C>A>B | | |  |
| Optimum level | A2 | B3 | C1 |  | Optimum level | A3 | B1 | C1 |  |
| Optimal combination | A2B3C2 | | |  | Optimal combination | A3B1C1 | | |  |

**Table 5. Main effect analysis of the abundance and diversity of bacteria and fungi in different agronomic practices.**

|  | Bacteria | | | | Fungi | | | |
| --- | --- | --- | --- | --- | --- | --- | --- | --- |
|  | Chao1 index | ACE index | Simpson index | Shannon index | Chao1 index | ACE index | Simpson index | Shannon index |
| Den | ** | ** | ns | ns | ns | ns | ** | ** |
| Irr | ** | ** | ns | * | ns | ns | * | ** |
| Fer | ns | ns | ns | ns | * | * | * | * |

Den: planting density; Irr: irrigation levels; Fer: fertilizer application: "*" and "**" denote significant differences at (*P* < 0.05) and (*P* < 0.01) levels, respectively, and ns denotes no significant difference.
